# Delayed Diagnosis: Adult-Onset Still's Disease Initially Mistaken for Tuberculosis in a Nigerian Man

**DOI:** 10.7759/cureus.110192

**Published:** 2026-06-03

**Authors:** Kate Sheridan, Zeeshan Subhani, Imran Patel

**Affiliations:** 1 Internal Medicine, Royal Preston Hospital, Preston, GBR; 2 General Medicine, Royal Preston Hospital, Preston, GBR

**Keywords:** adult-onset still's disease (aosd), extrapulmonary tuberculosis (eptb), hemophagocytic lymphohistiocytosis (hlh), macrophage activation syndrome (mas), race bias, race inequities

## Abstract

Adult-onset Still's disease (AOSD) is a rare autoimmune disorder characterised by fever, rash, joint pain, and elevated ferritin levels. It is extremely difficult to diagnose due to its overlap with other autoimmune and infective causes. If left untreated, systemic inflammation can rarely trigger an exaggerated immune response, leading to secondary haemophagocytic lymphohistiocytosis (HLH) or macrophage activation syndrome (a secondary subtype of HLH). This case demonstrates AOSD complicated by HLH in a Nigerian man in his 30s with fever, shortness of breath, night sweats, and joint pain, where the working diagnosis for weeks of admission was tuberculosis. Investigations suggested a lymphadenopathic infection; however, poor response to broad-spectrum therapies and repeated negative cultures eventually led to the consideration of AOSD. This patient developed high ferritin levels, pancytopenia, hypofibrinogenaemia, and hypertriglyceridaemia with both liver and kidney failure. This patient was taken to critical care with renal and liver dysfunction. He improved with aggressive steroid therapy and was discharged from hospital.

## Introduction

Adult-onset Still’s disease (AOSD) complicated by haemophagocytic lymphohistiocytosis (HLH) in a Nigerian man in his 30s with fever, shortness of breath, night sweats and joint pain, where the initial diagnosis was tuberculosis. Investigations suggested a lymphadenopathic infection; however, poor response to broad-spectrum therapies eventually led to the consideration of AOSD.

AOSD is an auto-inflammatory condition characterised by regular and intermittent high fevers, arthralgia or arthritis, and a typical ‘salmon-pink’ rash. Other symptoms can include sore throat, myalgia, and lymphadenopathy/organomegaly [1]. In comparison, tuberculosis can present with cough, haemoptysis, and chest pain. Still, it may also present with more general symptoms such as loss of appetite, unexpected weight loss, night sweats, fever, fatigue, and arthralgia [2].

There is little research into the epidemiology of AOSD; however, its annual incidence is estimated at 0.14-0.6 per 100,000. It is more common in women and has a bimodal age distribution (16-25 years, then 35-45 years) [3]. However, in 2024, the annual incidence of tuberculosis was 9.4 per 100,000 in the United Kingdom [4]. Given the overlap in symptoms between these two conditions and the increased prevalence of tuberculosis, cases of AOSD may not be considered as the most likely differential. Furthermore, the cutaneous manifestations of AOSD may present differently in people of darker skin colours, making it even harder to diagnose in this case.

The exact pathophysiology of AOSD is unknown, and it is thought to be a mix of infection and genetics. No definitive infectious source has been found. Numerous cytokines (IL-6, IL-18, IFN-γ, TNF-α, macrophage-CSF) are thought to contribute to the development of AOSD [5]. To fulfil the criteria for AOSD, the patient must meet five or more of the Yamaguchi criteria, including two or more major criteria. Major criteria include intermittent fever >39°C for one week or longer, arthralgia for >2 weeks, a salmon-like rash, and WBC >10 × 10⁹/L with 80% neutrophils. Minor criteria include sore throat, splenomegaly, liver abnormalities, and negative ANA and RF [6].

## Case presentation

A normally fit and well Nigerian man in his 30s with no recent foreign travel presented to the emergency department with a one-week history of shortness of breath, chest pain, and fever. He also had a one-week history of joint pain in his left knee and both ankles, which were swollen with reduced range of movement. This was accompanied by a persistent purple rash on his right shoulder, which deviates significantly from the expected ‘salmon-pink’ rash of AOSD and complicated the diagnosis.

On day 1, his blood tests showed significant leukocytosis (WBC: 23.22 × 10⁹/L, neutrophils: 20.82 × 10⁹/L) and thrombocytosis (470 × 10⁹/L). A chest X-ray was normal, as were bilateral knee X-rays. On day 7, he tested negative for COVID-19, flu, HIV, hepatitis, connective tissue diseases, vasculitis, and rheumatological antibodies. From day 5 until day 18, he remained on broad-spectrum antibiotics to cover for bacterial infection.

Investigations

**Figure 1:**
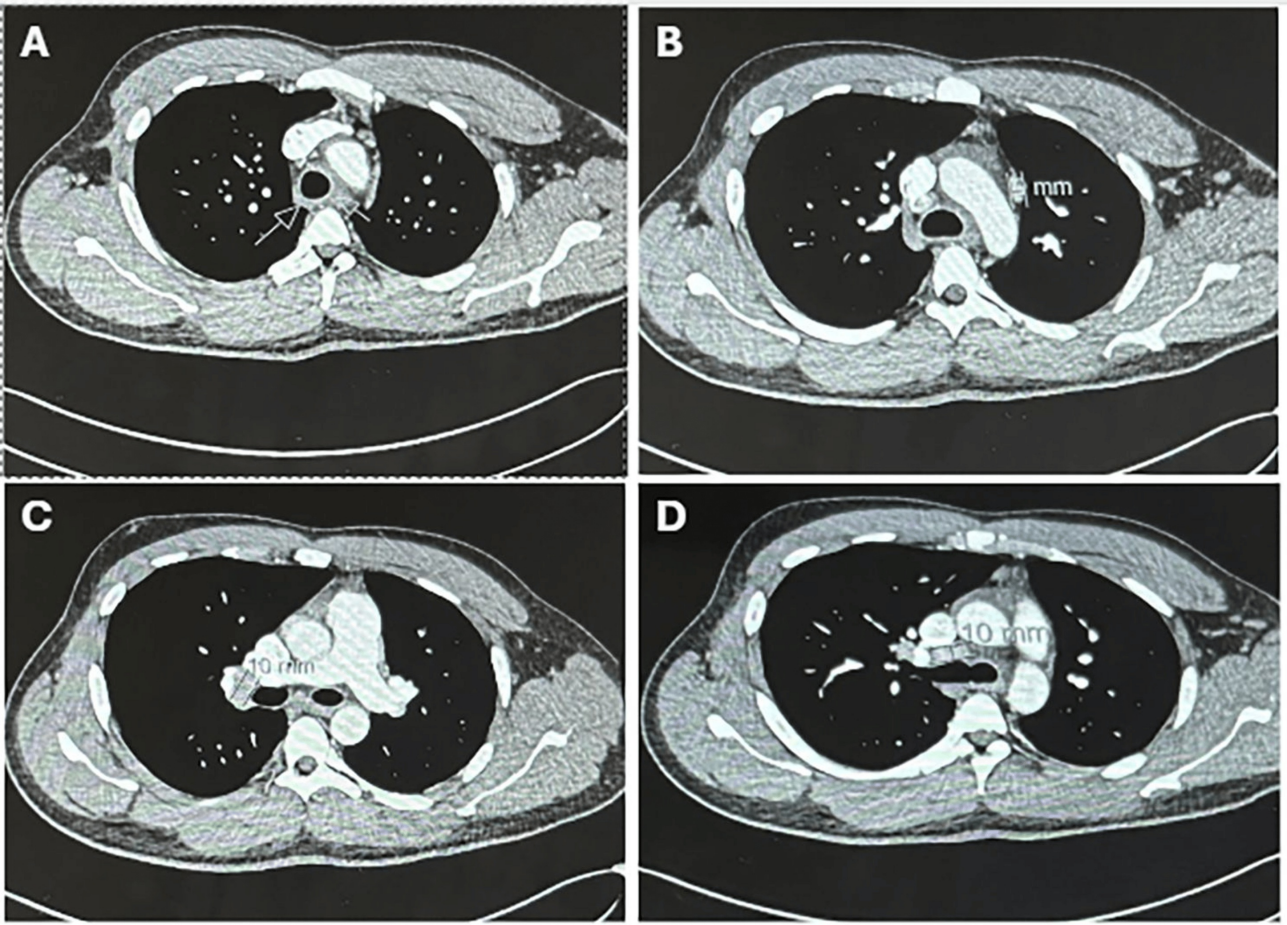
Soft tissue thickening around the trachea and upper half of the oesophagus (B) and mediastinal and hilar lymphadenopathy (B, C, D)

**Figure 2:**
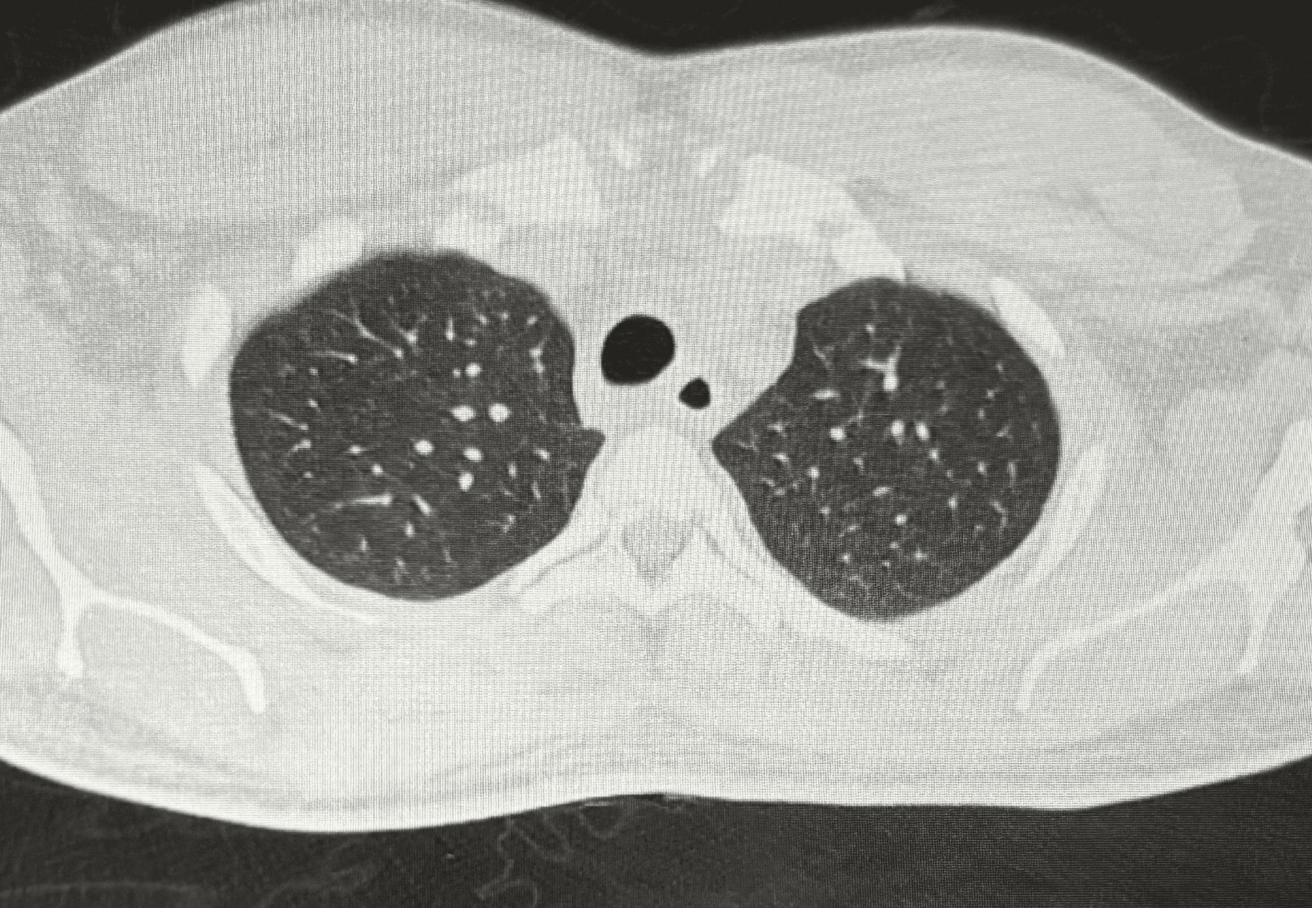
CT with contrast performed to rule out oesophageal perforation CT: computed tomography

A CT thorax, abdomen, and pelvis was performed on day 4 due to diagnostic uncertainty. This showed scattered nodal enlargement in the mediastinum and both pulmonary hila, with non-specific soft-tissue opacification around the trachea and the upper half of the oesophagus (Figure 1). Repeat CT on day 5 showed no evidence of oesophageal perforation (Figure 2).

**Figure 3:**
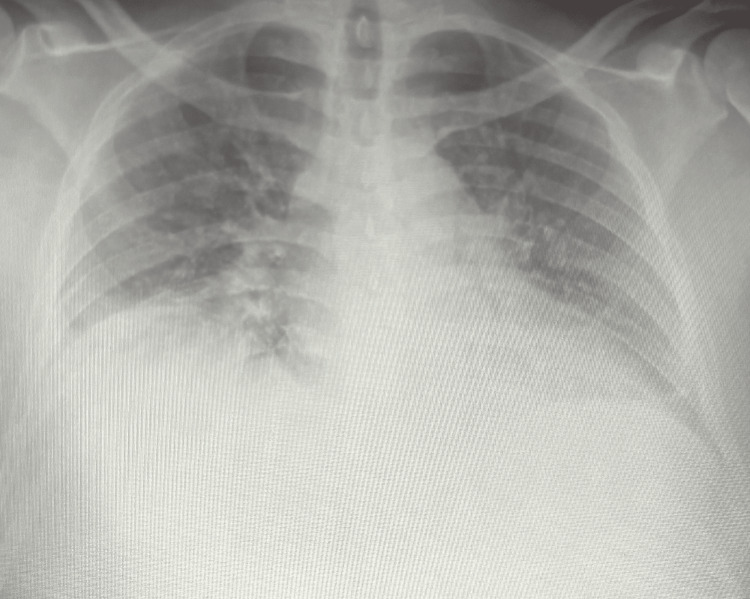
Chest X-ray showing bibasal consolidation

**Figure 4:**
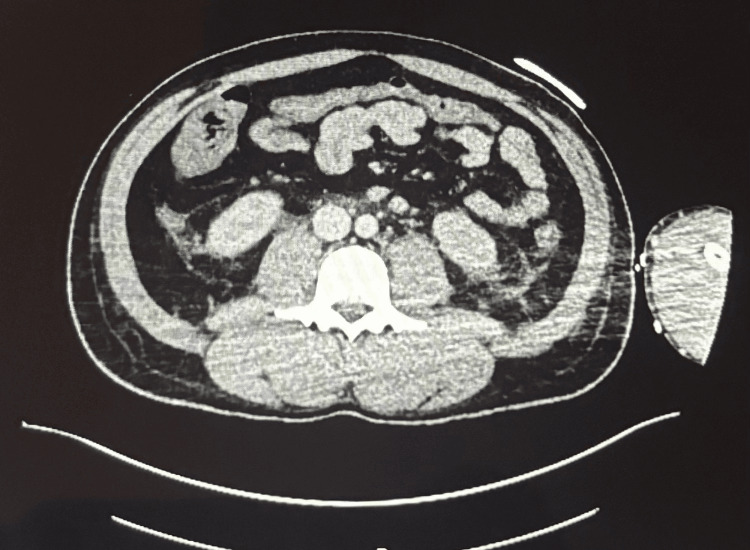
CT abdomen showing mild acute colitis from the ascending to descending colon CT: computed tomography

Further imaging and investigations were mostly normal, aside from a chest X-ray showing bibasal consolidation (Figure 3), a CT abdomen showing mild acute colitis (Figure 4), and an echocardiogram showing a thin, loculated pericardial effusion.

On day 5, further blood tests were performed. This patient had three negative mycobacterial cultures, a negative malaria screen, a negative EBV screen, and a negative cytomegalovirus screen. Blood film on day 12 showed early immature myeloid cells, neutrophil vacuolation, and a left-shifted neutrophil distribution. The first suggestion of Still’s disease came on day 13 from the respiratory team, who suggested ferritin, LDH, and daily laboratory monitoring for HLH. Notable positive tests included elevated LDH, elevated triglycerides, ferritin that increased from 25,000 to over 100,000 within 48 hours, elevated procalcitonin, elevated AST, low fibrinogen, and progressively worsening liver function. Three acid-fast bacillus tests on sputum were negative for tuberculosis, as were two malaria samples (Table 1).

**Table 1:** Summary of both inflammatory and autoimmune test results HIV: human immunodeficiency virus, PCR: polymerase chain reaction, ESR: erythrocyte sedimentation rate, CTD: connective tissue disease, ANCA: anti-neutrophil cytoplasmic antibodies, GBM: glomerular basement membrane, IgG: immunoglobulin G

Pathology	Result
Blood culture	No growth
Mycobacterium culture	Negative
Hepatitis B and C	Negative
HIV	Negative
Malaria	Negative
Epstein-Barr virus	Negative
Cytomegalovirus	Negative
Chlamydia PCR	Negative
Sputum culture	No growth
Urinary antigen screen	No growth
C3/C4 complement	C3 raised
Anti-phospholipid	Negative
Rheumatoid factor	Negative
ESR	113
CTD screen	Negative
ANCA	Negative
Anti-GBM	Negative
Immunoglobulins	Raised IgG
Paraproteins	Not detected
Treponemal antibody	Negative

By day 14, the multidisciplinary team agreed that Still’s disease was a strong possibility and decided to trial intravenous steroids. Unfortunately, the patient deteriorated into multi-organ failure and was transferred to critical care on day 15, where he was intubated and ventilated.

**Figure 5:**
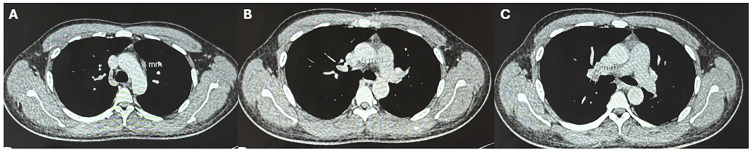
Prevascular lymph node reduction from 8 mm to 3 mm (A), precarinal lymph node reduction from 10 mm to 5 mm (B), and right hilar lymph node from 11 mm to 9 mm (C)

On day 17, a bronchoalveolar lavage was performed with samples sent for tuberculosis and Pneumocystis pneumonia, both returning negative, further supporting Still’s disease as the most likely diagnosis. On day 20, a bone marrow biopsy showed no evidence of lymphoma, only reactive changes. The patient was stepped down from critical care to the renal ward on day 23. An endoscopy was performed on day 29 due to oesophageal inflammation seen on CT, but was normal. On day 31, a repeat CT chest showed that the right hilar and mediastinal lymph nodes had decreased in size and were likely reactive (Figure 5).

It is worth noting that multiple specialties were consulted regarding the non-specific imaging findings, and they collectively decided against further invasive investigations. The cardiology team felt that the small rim of pericardial effusion was related to the underlying disease process and would resolve with anti-inflammatory therapy. The gastroenterology team also felt that the colitis seen on CT was likely secondary to the same process and would resolve with anti-inflammatory therapy. The respiratory team felt that an endobronchial ultrasound was not required, given that the bone marrow biopsy showed no evidence of lymphoma and that CT imaging demonstrated partial resolution of lymph node enlargement. The renal team felt that a renal biopsy was unnecessary, as the patient’s renal function had recovered and continued to improve even after withdrawal of dialysis.

Diagnosis

Differentials included mediastinitis, tuberculosis, and lymphoma. Tuberculosis and other bacterial/viral infections were excluded through screening prior to diagnosis. AOSD was considered only near the end of admission. By that point, the patient met three of the major Yamaguchi criteria (persistent fever, two weeks of arthritis, neutrophilia) and most of the minor criteria (sore throat, lymphadenopathy, negative RF and ANA, and deranged liver function tests). By the time of diagnosis, the patient also fulfilled criteria for HLH with raised ferritin, low fibrinogen, high triglycerides, and hyponatraemia. His H-score, calculated on day 15 when all blood results were available, was 192, exceeding the optimal diagnostic cutoff of 169 (Table 2) [7].

**Table 2:** Breakdown of H-score AST: aspartate aminotransferase

Criteria	Result
Known underlying immunosuppression	No - 0 points
Temperature	41.2 °C (>39.4) - 49 points
Organomegaly	No - 0 points
Number of cytopenias	1 lineage, platelets <110 - 0 points
Ferritin	>100,000 (>6,000) - 50 points
Triglycerides	2.87 (1.5-4) - 44 points
Fibrinogen	1.9 (<2.5) - 30 points
AST	1,801 (>30) - 19 points
Haemophagocytosis features on bone marrow aspirate	No - 0 points

Treatment

**Figure 6:**
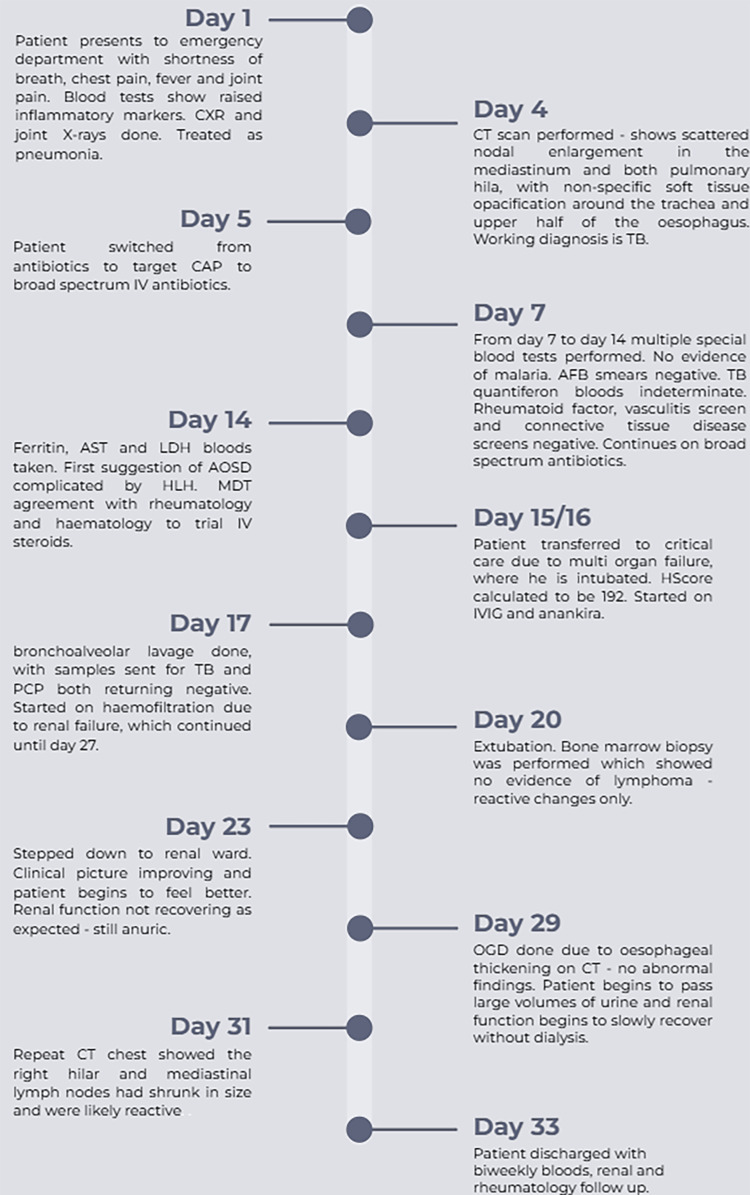
Timeline showing events of admission CXR: chest X-ray, CT: computed tomography, TB: tuberculosis, CAP: community-acquired pneumonia, AFB: acid-fast bacilli, AOSD: adult-onset Still's disease, HLH: haemophagocytic lymphohistiocytosis, MDT: multidisciplinary team, IV: intravenous, H-score: hemophagocytic syndrome diagnostic score, IVIG: intravenous immunoglobulin, PCP: Pneumocystis pneumonia, OGD: oesophago-gastro-duodenoscopy, AST: aspartate aminotransferase, LDH: lactate dehydrogenase

The patient was started on high-dose intravenous methylprednisolone on day 14, followed by anakinra and intravenous immunoglobulin, with prophylactic aciclovir and amphotericin from day 16. The patient’s inflammatory markers began to trend down, and his liver function gradually improved (Table 3). He received hemofiltration from day 17 until day 27 and was extubated on day 20, before being stepped down to the renal ward on day 23. His renal function improved after hemofiltration and continued to improve after it was discontinued. A general overview of the patient’s admission is shown in Figure 6. It is worth noting that the increase in WBC on day 1 post-treatment was likely due to steroid-induced neutrophilia, a recognised side effect of corticosteroid therapy.

**Table 3:** Progression of HLH bloods throughout admission WBC: white blood cell, Hb: haemoglobin, ALT: alanine aminotransferase, AST: aspartate aminotransferase, HLH: haemophagocytic lymphohistiocytosis

Test	Pre-treatment	Day 1 post-treatment	Day 3 post-treatment	Day 5 post- treatment	Day 7 post-treatment	Discharge	Reference range
WBC	16.63	20.54	13.86	6.54	7.50	4.21	4-11 x10^9^/L
Neutrophils	15.46	19.18	13.00	5.24	5.28	3.00	1.5-7.5 x10^9^/L
Hb	103	91	93	91	87	73	130-170 g/L
Platelets	462	305	46	60	85	284	150-450 x10^9^/L
Ferritin	24,924	>100,000	>100,000	96,438	31,741	3,975	24-340 ug/L
Fibrinogen	>5	4.5	1.9	1.9	1.9	3.4	1.5-4 g/L
Triglycerides	1.07	2.87	5.83	11.20	9.48	2.71	<1.7 mmol/L
Sodium	131	130	127	129	128	149	135-145 mmol/L
ALT	501	535	854	1348	618	62	7-56 U/L
AST	-	1801	1538	743	116	38	8-48 U/L

The patient was discharged on day 33 and will be followed up in the renal and rheumatology clinics for the foreseeable future. It is expected that his renal function will eventually return to baseline (eGFR >90). He was discharged on 100 mg subcutaneous anakinra twice daily, to be reduced to 100 mg once daily after five days and then continued long-term, along with 60 mg oral prednisolone once daily, also for tapering. He will undergo HLH blood tests bi-weekly.

The duration of these treatments depends on how long it takes to achieve complete remission. Most patients can eventually discontinue treatment. There are no established guidelines for tapering therapy, but it is usually recommended after patients have been in remission for three months. Patients should still undergo regular monitoring of full blood count, renal function, liver function, and ferritin.

## Discussion

There are three main clinical courses of AOSD: self-limiting (single episode with sustained remission), intermittent (recurrent flares with remissions), and chronic (persistent symptoms lasting more than one year). Life-threatening complications include pleuritis, pericarditis, liver failure, markedly elevated ferritin levels, and even death [8].

HLH is a life-threatening syndrome caused by a dysregulated inflammatory response due to overactivity of antigen-presenting cells. It is divided into primary (familial) HLH and secondary HLH. Familial HLH is an autosomal recessive condition. Secondary HLH is usually associated with infectious diseases, autoimmune diseases, malignancy, immunosuppression, stem cell or solid organ transplantation, HIV, and metabolic disorders [9].

The prognosis of AOSD is generally good if treated; however, evidence shows that up to 75% of patients experience relapse [10]. HLH has a poor prognosis, with an estimated median survival of less than two months if untreated. Diagnosis is often delayed due to non-specific clinical findings and symptom overlap with other conditions [11].

Despite overall treatability, AOSD still carries significant morbidity and mortality in cohort studies. Although there are few large-scale studies describing risk factors for adverse outcomes, a US study of over 5,000 patients found that individuals of Asian origin had higher odds of in-hospital mortality compared with white patients [12]. There are very few case reports of Nigerian patients with AOSD and HLH, and limited research into how race may influence diagnosis, treatment, and prognosis. Some studies suggest increased pulmonary involvement in individuals of African descent, while cutaneous manifestations may differ from the typical 'fleeting, salmon-pink rash' described in lighter skin tones [13]. Flagellate-patterned eruptions, hyperpigmentation, and persistent rashes are atypical presentations more frequently reported in lighter-skinned populations, but there is limited guidance on how these present in darker skin types [13].

## Conclusions

This case report highlights the diagnostic complexity of both AOSD and HLH, particularly in people with darker skin tones, in whom cutaneous manifestations form part of the diagnostic criteria. The rapid progression of HLH and its high mortality if left untreated highlight the need for prompt diagnosis and a high index of suspicion, especially in patients with different skin tones who may not present in a 'typical' way. Ultimately, this case underscores the need for ongoing education on AOSD, HLH, and dermatological conditions in people with darker skin.
